# Dysphagia assessment in ischemic stroke after mechanical thrombectomy: When and how?

**DOI:** 10.3389/fneur.2022.1024531

**Published:** 2022-11-23

**Authors:** Sriramya Lapa, Elisabeth Neuhaus, Elena Harborth, Vanessa Neef, Helmuth Steinmetz, Christian Foerch, Sarah Christina Reitz

**Affiliations:** ^1^Department of Neurology, University Hospital Frankfurt, Goethe University, Frankfurt am Main, Germany; ^2^Institute of Neuroradiology, University Hospital Frankfurt, Goethe University, Frankfurt am Main, Germany; ^3^Department of Anesthesiology, Intensive Care Medicine and Pain Therapy, University Hospital Frankfurt, Goethe University, Frankfurt, Germany

**Keywords:** dysphagia, stroke, thrombectomy, aspiration, endovascular treatment, fiberoptic endoscopic evaluation of swallowing (FEES)

## Abstract

**Background:**

Dysphagia is a frequent symptom in acute ischemic stroke (AIS). Endovascular treatment (EVT) has become the standard of care for acute stroke secondary to large vessel occlusion. Although standardized guidelines for poststroke dysphagia (PSD) management exist, they do not account for this setting in which patients receive EVT under general anesthesia. Therefore, the aim of this study was to evaluate PSD prevalence and severity, as well as an appropriate time point for the PSD evaluation, in patients undergoing EVT under general anesthesia (GA).

**Methods:**

We prospectively included 54 AIS patients undergoing EVT under GA. Fiberoptic Endoscopic Evaluation of Swallowing (FEES) was performed within 24 h post-extubation in all patients. Patients presenting significant PSD received a second FEES-assessment to determine the course of dysphagia deficits over time. Dysphagia severity was rated according the Fiberoptic Dysphagia Severity Scale (FEDSS).

**Results:**

At first FEES (FEES 1) assessment, performed in the median 13 h (IQR 5–17) post-extubation, 49/54 patients (90.7%) with dysphagia were observed with a median FEDSS of 4 (IQR 3–6). Severe dysphagia requiring tube feeding was identified in 28/54 (51.9%) subjects, whereas in 21 (38.9%) patients early oral diet with certain food restrictions could be initiated. In the follow up FEES examination conducted in the median 72 h (IQR 70–97 h) after initial FEES 34/49 (69.4%) patients still presented PSD. Age (*p* = 0.030) and ventilation time (*p* = 0.035) were significantly associated with the presence of PSD at the second FEES evaluation. Significant improvement of dysphagia frequency (*p* = 0.006) and dysphagia severity (*p* = 0.001) could be detected between the first and second dysphagia assessment.

**Conclusions:**

PSD is a frequent finding both immediately within 24 h after extubation, as well as in the short-term course. In contrast to common clinical practice, to delay evaluation of swallowing for at least 24 h post-extubation, we recommend a timely assessment of swallowing function after extubation, as 50% of patients were safe to begin oral intake. Given the high amount of severe dysphagic symptoms, we strongly recommend application of instrumental swallowing diagnostics due to its higher sensitivity, when compared to clinical swallowing examination. Furthermore, advanced age, as well as prolonged intubation, were identified as significant predictors for delayed recovery of swallowing function.

## Introduction

Dysphagia is a frequent symptom of acute ischemic stroke (AIS), with a reported prevalence of up to 78% (1). It is associated with aspiration pneumonia, prolonged hospital stays, as well as increased mortality and morbidity (2). Early identification and treatment of patients with post-stroke dysphagia (PSD) reduces the risk of pulmonary, nutritional and hydration related complications, and shortens hospitalization (3). Hence, the current standard of stroke care requires (a) early screening for PSD before the administration of food, drink or oral medication followed by (b) a detailed swallowing evaluation by a speech and language therapist (SLT) in those patients with suspected dysphagia, in order to determine dysphagia severity and the need for further instrumental swallowing assessment (2, 4, 5).

Over the past years, endovascular treatment (EVT) has become the standard of care for acute stroke secondary to large vessel occlusion (LVO). Multiple studies have demonstrated reduced rates in mortality and improvements in National Institutes of Health Stroke Scale (NIHSS) and Modified Rankin Scale (mRS) scores for patients following EVT (6–8). Considering the increasing use of EVT, the medical team, including SLTs, must deal with this recent treatment for acute ischemic stroke. In contrast to thrombolytic therapy, EVT requires either conscious sedation or general anesthesia (GA), with both temporarily compromising swallowing physiology (9–13). In contrast to conventional stroke treatment, EVT requires the use of sub-anesthetic levels of drugs which are known to cause pharyngeal dysfunction due to depressed swallowing reflex and increasing latency to initiate swallow, even after recovery of consciousness (11, 14).

Furthermore, the presence of an endotracheal tube could lead to dyssynchronous breathing and swallowing, as well as to mechanical irritations of the endo-laryngeal mucosal tissue, resulting in diminished laryngeal sensory function. Beyond the impact of the underlying disease, ICU procedures (e.g., intubation, mechanical ventilation, or sedation) themselves must be considered as risk factors for the development of dysphagia. Hence, high dysphagia rates with up to 40% of patients presenting aspiration post-extubation are also observed in non-neurological ICUs [e.g., medical-surgical, and cardiac ICU (15–17)].

Moreover, patients receiving EVT suffer from severe stroke with reduced cerebral blood flow in the brain regions that are critical for swallowing control, hereby significantly increasing the risk for PSD (18, 19).

Unfortunately, studies exploring the effect of reperfusion therapy on swallowing are scarce, with little to no research focusing on the impact of EVT on PSD (20, 21). Hence, the influence of GA, EVT or the stroke itself on swallowing physiology remains unclear. According to clinical guidelines PSD assessment should be performed quickly after emergent stroke workup, regardless of stroke severity, in fact absolutely within 24 h. However, data concerning the appropriate time point for dysphagia assessment in patients receiving sedation and/or endotracheal intubation for EVT is lacking. In general, swallowing evaluation is often delayed at least 24 h following extubation, assuming that swallowing function improves over time. This might delay drug administration and increase the risk of dehydration and malnutrition. Plus, delayed dysphagia evaluation is known to be associated with a higher risk of complications like pneumonia (22). Therefore, we tried to identify an appropriate time point for dysphagia assessment in this specific stroke-population undergoing GA.

Recommendations when (e.g., how many hours after extubation) and how [Clinical swallowing examination (CSE) or instrumental examination] to evaluate PSD following EVT are vital to support clinical decision making for optimal dysphagia management.

The aim of this explorative study was to evaluate PSD prevalence and severity, as determine an appropriate time for PSD evaluation in patients undergoing EVT under GA. Lastly, we tried to identify clinical variables that would enable us to estimate short-term dysphagia outcome.

## Materials and methods

### Data availability

The data that support the findings of this study are available upon reasonable request to the corresponding author.

### Patients and study design

This study was conducted at the University Hospital Frankfurt am Main, Goethe University, which has an EVT-capable stroke center with 24/7 thrombectomy capacity. Following our in-house standards, all EVT procedures were performed under general anesthesia (GA). For mechanical thrombolysis, either mechanical aspiration or a stent retriever device was used. Eligible patients received intravenous recombinant tissue plasminogen activator before thrombectomy.

From June 2019 until March 2020, patients with acute intracranial LVO, who underwent EVT at our department, were screened for study eligibility. Inclusion criteria were: (1) Proximal occlusion of intracranial artery (CT-A or TOF MRI), (2) undergoing endovascular treatment, and (3) FEES assessment within 24 h post-extubation.

Exclusion criteria: (1) history of pre-existing dysphagia or any concomitant disease likely to cause dysphagia, (2) spontaneous recanalization during angiography, (3) palliative extubation, (4) discharged upon a ventilation, or (5) no informed consent.

Informed consent was obtained from all patients or their legal representative. The nature of the study was approved by the local ethics committee of the Goethe University Hospital Frankfurt (approval no. 19-262), and was conducted according to the principles of the Declaration of Helsinki.

### Dysphagia assessment

All patients included in this study received a bedside clinical swallowing evaluation (CSE) by an SLT at the earliest 2 h, but within 24 h, post-extubation. Patients showing one or more symptoms predictive for PSD (i.e., failing of a simple water swallowing test, moderate dysarthria/aphasia, facial palsy and ≥NIHSS 5) underwent FEES-examination subsequently (FEES 1) (23, 24).

Each examined patient was classified according to a 6-point scoring system,—the Fiberoptic Endoscopic Dysphagia Severity Scale (FEDSS)—ranking dysphagia severity with 1 scoring best and 6 being worst. Patients were categorized as mildly dysphagic (1–3) and severely dysphagic (4–6, 25).

Dysphagia was deemed to be present if one or more of the following signs of swallowing dysfunction were detected, during endoscopic swallowing examination: disturbed management of secretions (i.e., pooling or aspiration of saliva), penetration or aspiration of any food consistency, relevant pharyngeal food residue after the swallow, or delayed swallowing reflex (26).

Additionally, laryngeal injuries were assessed according to a previously defined injury scheme, which reads as 0 = no injury present, 1 = soft tissue injury (e.g., edema, erythema), 2 = hematoma, ulceration, fibrin without glottic narrowing, mass lesion, granulation, 3 = stenosis, stenosis with glottic narrowing, hypomobility/immobility of the vocal folds and/arytenoids complex (12).

Patients presenting significant dysphagia requiring supplemental feeding (FEDSS 3–6) underwent a second FEES (FEES 2), 72 h after the first FEES (FEES1) was conducted, to evaluate the course of dysphagia. All dysphagic patients received daily dysphagia treatment and re-evaluation of swallowing function by an SLT, until they were discharged or returned to full oral diet with any food restrictions. Determination of oral diet was according to the following standardized in-house guidelines.

Based on the nutrition recommendation derived from the FEDSS, an appropriate oral diet was chosen for each patient based on the first FEES findings (FEES 1). Hence, early oral feeding with a specially adapted diet not requiring professional supervision was started in all patients presenting mild dysphagia [FEDSS score (1) soft solid food and liquids, score, (2) pureed food and liquids, score, and (3) pureed food and parenteral application of liquids].

Dysphagic patients requiring supplemental feeding (FEEDS 3–6) at the first endoscopic evaluation (FEES 1) were investigated a second time with FEES (FEES 2). Based on the second FEES results, an appropriate oral diet was chosen for each patient containing all textures declared as safe to swallow (Score 1–3 on the Rosenbek's Penetration-Aspiration-Scale) (27).

In patients with full oral diet (FEDSS 1–2) the ability to swallow solid texture was daily re-evaluated by the treating SLT, for disturbed oral preparatory phase or aspirations signs.

### FEES equipment and protocol

FEES equipment consisted of a 3.9-mm-diameter video rhinolaryngoscope (RS1/RX1 Orlvision, Germany), a 150 W light source for endoscopic application (rp-150), a camera (rpCam62, S/N), a color monitor (7′-TFT-EIZO, 1,500:1) and a video recorder (1/2” CCD-camera, rp Cam62).

FEES examination was performed by two SLTs following a previously described protocol starting with the recording of any event of saliva pooling or aspiration (28). Afterwards all patients received different food consistencies, starting with semisolid (pudding), liquid (water), followed by solid texture (bread). Each consistency was regularly tested 3 times, only in case of penetration to the vocal folds and/or aspiration without total ejection of the material or in case of severe residues (Grade 4 according to the Yale Pharyngeal Residue Severity Scale), we did refrain from further examination with the respective texture (29). All food were blue dyed for better contrast with pharyngeal and laryngeal mucosa.

### Clinical variables

We assessed age, gender, vascular risk factors, stroke etiology, occlusion side and localization, duration of ventilation, and administration of IVT as pre-treatment medication, before EVT. Response of mechanical thrombolytic therapy was rated according to the thrombolysis in cerebral infarction (mTICI) grading system (30). Stroke severity, in terms of the score on the National Institutes of Health Stroke Scale (NIHSS), was evaluated at the time of admission, the time point of the first and second FEES assessments, and at discharge. The diagnosis of pneumonia was based on ≥3 of the following features: fever (>38°C), productive cough, abnormal respiratory examination, abnormal chest radiograph, white blood cell count >12,000/ml, or isolation of a relevant pathogen, or the use of antibiotics (19). Degree of disability prior to stroke was estimated at time of admission and eventually assessed at discharge using the modified Rankin Scale (mRS).

### Brain imaging assessment

Brain images were independently rated by an experienced blinded neuroradiologist (EN). Ischemic changes were determined on the first cranial CT or MRI scan after EVT. Infarction within the MCA territory was evaluated according to the Alberta stroke program early CT score (ASPECTS) (31). In brief, the MCA territory in both hemispheres was divided into 10 regions, each representing 1 point on the 10-point ASPECT score. A single point was subtracted for an area of ischemic changes on CT. One patient with basilaris artery occlusion received cranial magnetic resonance imaging for assessment of infarction.

### Interrater reliability

All videos (first and second FEES-assessment) were independently scored by two raters (CN and EH), who were blinded to the patients and their medical conditions. For final analysis of the results, disagreement concerning the presence of dysphagia and the severity of dysphagia in terms of the FEDSS was discussed until consensus was reached.

The interrater reliability for the FEDSS were almost perfect at both assessment time points, with a κ-value of 0.93 (*p* < 0.001; first FEES) and a κ-value of 0.97 (*p* < 0.001; second FEES) respectively. Evaluation of laryngeal injury for both FEES assessments showed substantial level of agreement (κ-value 0.66, *p* < 0.001 for FEES 1 and κ-value 0.72, *p* < 0.001 for FEES 2) (32).

### Statistical analysis

Baseline characteristics data are presented as medians (interquartile ranges, IQR) or means (depending on the presence of normal-distribution, tested by quantile-quantile plots) or numbers with percentages, unless otherwise indicated. The statistical significance of differences between them were assessed *via* Wilcoxon-matched-pair test or Friedmann-test, depending on scale level and group. Pearson's square test was used for binary variables. Predictors for dysphagia were evaluated using binary logistic regression analysis (dependent variable dysphagia, covariates: age, sex, NIHSS at first FEES assessment (FEES 1), occlusion side right/left, ventilation time, lesion location [frontal operculum (M1), internal capsule (CI), insula cortex (I)]. Interrater reliability was analyzed for both FEES assessments using Cronbach's alpha.

The significance level was set to *p* < 05. Statistical analysis was performed with SPSS version 26.0 (IBM) and GraphPad Prism 9.0 (GraphPad Software).

## Results

**Figure 1:**
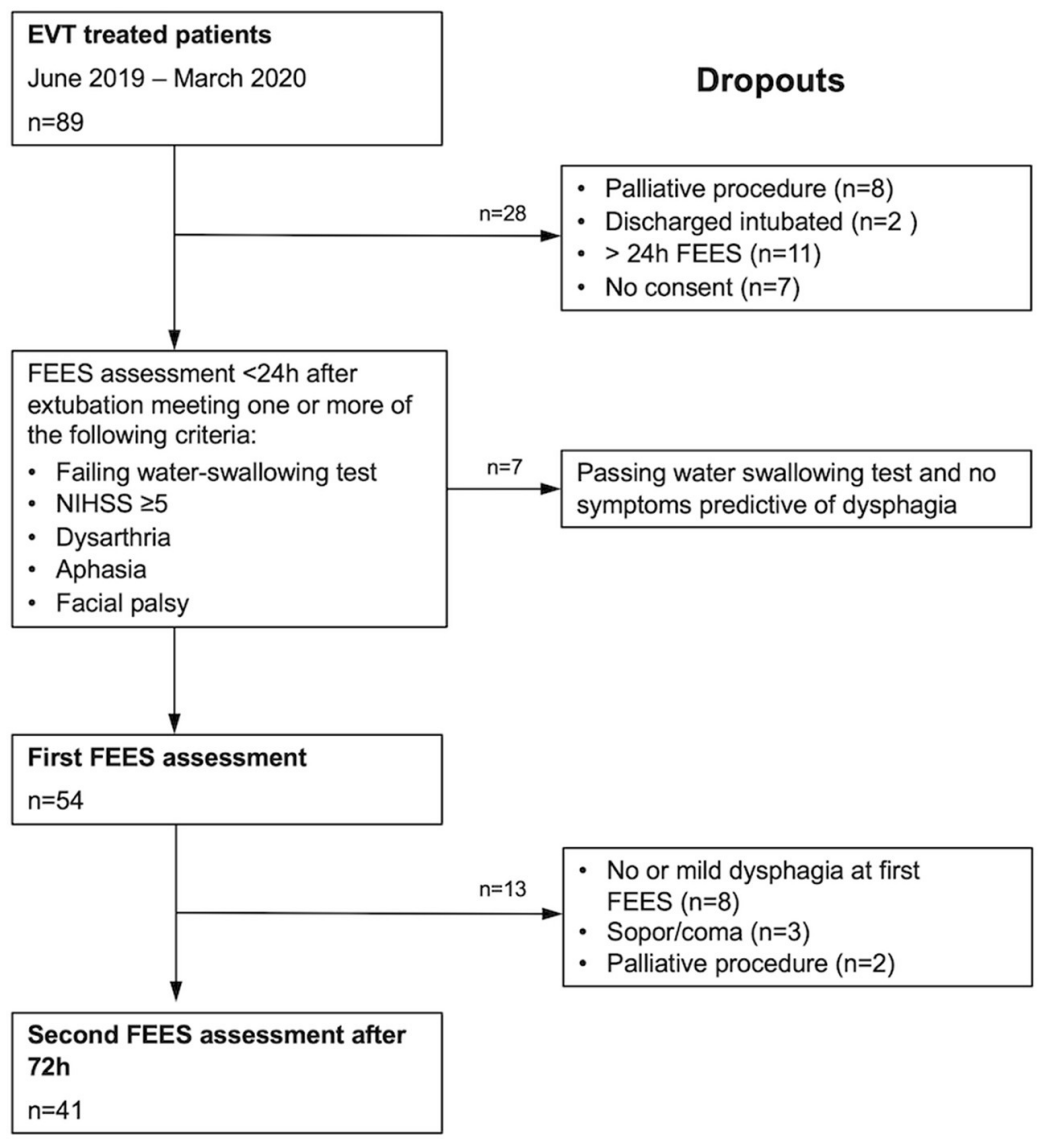
Consort diagram detailing the number of patients recruited into the study and reasons for dropout.

**Figure 2:**
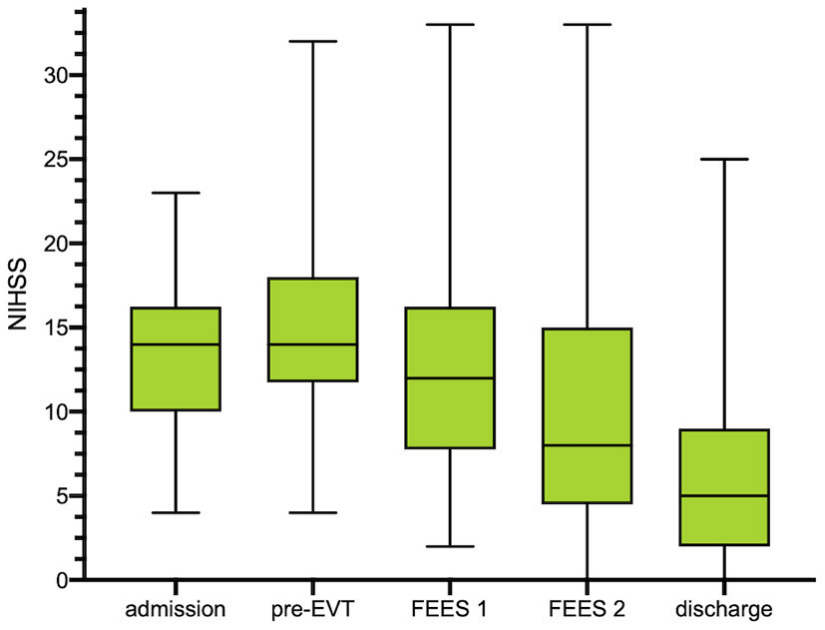
Box plots show the distributions of the National Institutes of Health Stroke Scale (NIHSS) scores at any clinical assessment: at pretreatment assessment, at d0 [after endovascular treatment (EVT)], at first FEES assessment (< 24 h after extubation) and at second FEES assessment. NIHSS is depicted in median with IQR as well as minimum and maximum for each time point. *No NIHSS at FEES 2 available due to reintubation (*N* = 1) or death (*N* = 3). FEES, fiberoptic endoscopic evaluation of swallowing; NIHSS, national institutes of health stroke scale; IQR, interquartile range.

A total of 89 patients with acute stroke to LVO were screened for eligibility (Figure 1). 54 patients (46% female; mean age 72 ± 13 years) meeting the inclusion criteria were enrolled in this study. 98% (*n* = 53) exhibited occlusion of the middle cerebral artery (right 55.6%, *n* = 30; left 42.5% *n* = 23) and 1.9% (1) of the basilar artery. Intravenous thrombolysis prior to EVT was performed in 33% of cases. 59.3% (*n* = 32) of the patients were secondarily transferred to our hospital for EVT. Successful recanalization of the occluded vessel could be accomplished in 88.8% (*n* = 48) of cases (TICI 2b and higher). Significant decrease in the NIHSS could be detected over the time period from admission [NIHSS of median 14 (IQR 10–16)] to discharge (NIHSS median 5, IQR 2–9; *z* = −4.87, *p* ≤ 0.001) (Figure 2). Baseline characteristics are depicted in Table 1.

**Table 1:** Demographic, clinical and baseline variables of the study population.

		**Dysphagia severity**		
	***n* (%) *n* = 54**	**FEDSS ≤ 3 *n* = 26**	**FEDSS ≥4 *n* = 28**	***p***
Age (year; mean ± SD)	72.1 ± 12.91	68.9 ± 12.87	75.0 ± 12.43	0.063
≥70	34 (63.0)	16 (61.5)	18 (64.3)	0.835
Length of stay	12 (8–16)	10.5 (8–15)	12 (9–19)	0.182
Female	25 (46.0)	13 (50.0)	12 (42.9)	0.599
Secondary transferred to EVT	32 (59.3)	12 (46.2)	20 (71.4)	0.059
IVT	33 (61.1)	20 (76.9)	13 (46.4)	0.022
General anesthesia	54 (100.0)	26 (100.0)	28 (100.0)	n.a.
Ventilation time (h; median, IQR)	5.6 (4–9)	4.9 (3–7)	6.9 (4–14)	0.018
**Risk factors**				
Smoking	8 (14.8)	3 (11.5)	5 (17.9)	0.514
Hypertension	50 (92.6)	22 (84.6)	28 (100.0)	0.031
Dyslipidemia	18 (33.3)	10 (38.5)	8 (28.6)	0.441
Diabetes	12 (22.2)	5 (19.2)	7 (25.0)	0.610
Prior stroke	13 (24.1)	4 (15.4)	9 (32.1)	0.150
Atrial fibrillation	23 (42.6)	9 (34.6)	14 (50)	0.253
Large–artery atheroslerosis	9 (16.7)	5 (19.2)	4 (14.3)	0.626
Cardioembolism	28 (51.9)	13 (50.0)	15 (53.6)	0.793
Other	17 (31.4)	8 (30.8)	9 (32.1)	0.914
**Occlusion side**				
Right	30 (55.6)	12 (46.2)	18 (64.3)	0.180
Left	23 (42.5)	13 (50.0)	10 (35.7)	0.289
Basilar	1 (1.9)	1 (3.8)	0 (0.0)	0.295
**Occlusion localization**				
ICA	6 (11.1)	3 (11.5)	3 (10.7)	0.923
Carotid–T	10 (18.5)	4 (15.4)	6 (21.4)	0.568
M1	33 (61.1)	15 (57.7)	18 (64.3)	0.620
M2	4 (7.4)	3 (11.5)	1 (3.6)	0.264
Basilar	1 (1.9)	1 (3.8)	0 (0.0)	0.295
**mTICI**				
0	4 (7.4)	1 (3.8)	3 (10.7)	0.336
1–2a	2 (3.8)	0 (0.0)	2 (7.2)	0.165
2b−3	48 (88.8)	25 (96.2)	23 (82.1)	0.102
**ASPECTS**				
Prior EVT (*n* = 51)	8 (7–9)	9 (7–10)	8 (7–9)	0.040
After EVT (*n* = 52)	6 (4–8)	7 (4–9)	5.5 (4–8)	0.211
**Symptom-onset to endovascular reperfusion** ***n*** **=** **50 (h; median, IQR)**	4.34 (3.5–6)	3.7 (3.1–6.2)	4.6 (3.7–6.3)	0.140
Prior EVT (*n* = 54)	14 (10–16)	13 (8–16)	14.5 (11–17)	0.196
FEES 1 (*n* = 54)	12 (8–16)	9 (7–12)	15 (11–19)	< 0.001
FEES 2 (*n* = 53)	8 (4.5–15)	5 (3–7.5)	13 (6.5–17)	< 0.001
At discharge (*n* = 51)	5 (2–9)	4 (2–6.5)	7.5 (4–13)	0.002
**FEDSS (median, IQR)**				
FEES 1	4 (3–6)	3 (2.5–3)	6 (5–6)	< 0.001
FEES 2	4 (2–6)	2 (1–3)	4 (3–6)	0.002
**mRS (median, IQR)**				
On admission	0 (0–1)	0 (0–1)	1 (0–4)	0.109
At discharge	4 (2-4)	3 (1–4)	4 (0–6)	0.002
**Pneumonia**	29 (53.7)	10 (38.5)	19 (67.5)	0.030

ASPECTS, alberta stroke program early CT score; EVT, endovascular treatment; FEDSS, fiberoptic endoscopic dysphagia severity scale; ICA, internal carotid artery; ICR, interquartile range; IVT, intravenous thrombolysis; NIHSS, national institute of health stroke scale; mTICI, thrombolysis in cerebral infarction; TOAST, trial of ORG 10172 in acute stroke treatment.

Twenty nine (53.7%) patients developed pneumonia, which occurred significantly more often in patients with NGT-feeding (FEDSS 4–6) than in patients with early oral diet (FEDSS 1–3) (χ^2^(1) = 6.58, *p* = 0.010). mRS increased significantly from status prior-stroke, with a median mRS of 0 (IQR 0–1) to a median of 4 at discharge (IQR 2–4; *z* = −5.94, *p* < 0.001). Three patients died after being included, but before second FEES assessment.

### Radiological findings

Of the 54 patients 31 (57.4%) presenting basal ganglia infarction (22 caudatus nucleus; 28 lentiform nucleus), the insular cortex was affected in 36 (66.6%) subjects, while in 15 (27.7%) cases damage to the internal capsule was observed. The cortical structures in the MCA territory were affected as follows: 26 (48.1%) M1, 27 (50%) M2, 11 (20.3%) M3, 16 (29.6%) M4, 15 (27.7%) M5, 19 (35.2%) M6. One patient presenting basilar artery occlusion received a brain MRI showing posterior cerebral artery territory infarction infarction (parieto-occipital and thalamus).

### First assessment (2–24 h after extubation) FEES 1

First assessment by an SLT, including instrumental swallowing examination, was performed in the median of 13 h (IQR 5–17) post-extubation. Of the 54 patients included in this study, 49 (90.7%) showed signs of dysphagia with FEES. 21 patients (38.9%) had mild to moderate swallowing impairment (FEDSS 1–3) whereas in 28 cases (51.9%) severe dysphagia was diagnosed (FEDSS 4–6).

**Figure 3:**
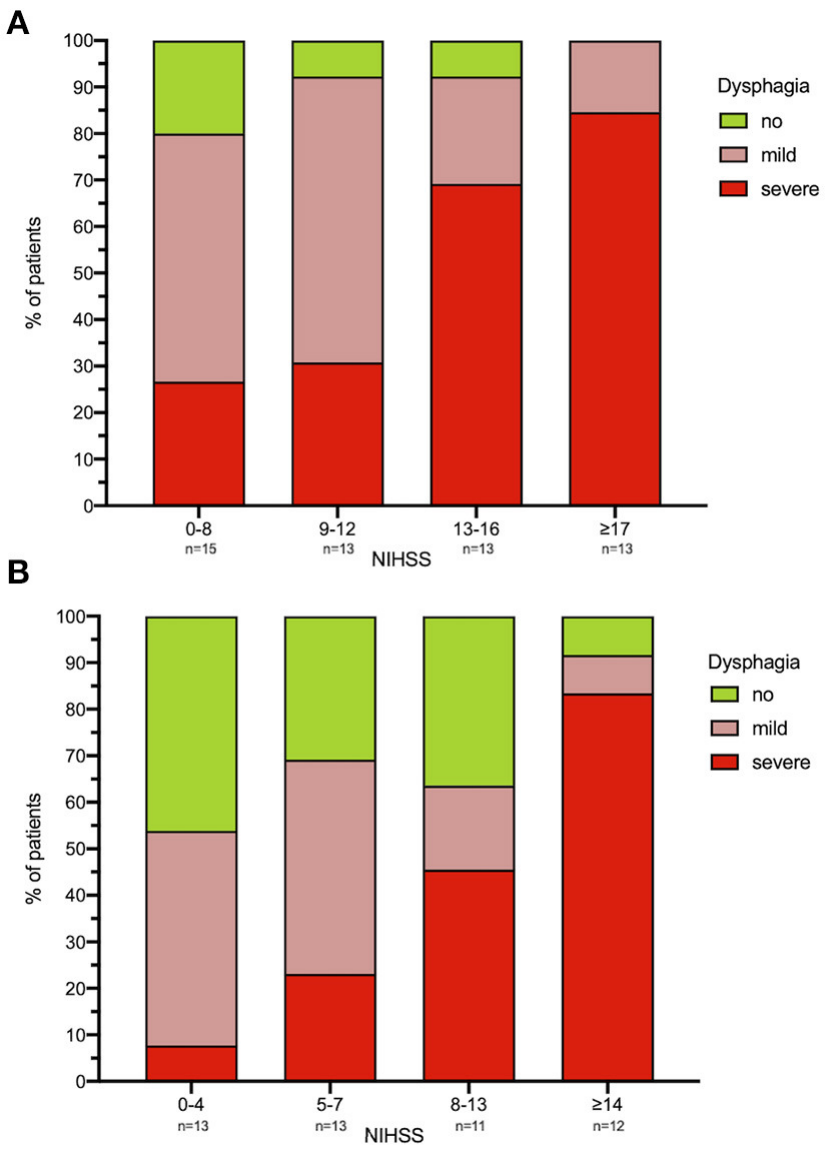
**(A,B)** Dysphagia severity in relation to NIHSS. For each time point [**(A)** FEES 1; **(B)** FEES 2] NIHSS was classified using a visual classification. Each column corresponds to ~25% of the study population. Dysphagia severity is defined as no dysphagia, mild dysphagia (FEDSS 1–3) and severe dysphagia (FEDSS 4–6). *N = 5 patients did not receive second FEES due to palliative procedure. FEES, fiberoptic endoscopic evaluation of swallowing; NIHSS, national institutes of health stroke scale; FEDSS, fiberoptic endoscopic dysphagia severity scale. FEES 1: NIHSS 0–8 *n* = 15, 9–12 *n* = 13, 13–16 *n* = 13, ≥17 *n* = 13. FEES 2: NIHSS 0–4 *n* = 13, 5–7 *n* = 13, 8–13 *n* = 11, ≥14 *n* = 12.

The median FEDSS was 4 (IQR 3–6). All patients with an NIHSS ≥ 17 had dysphagia, with 80% showing severely impaired swallowing function (FEDSS 4–6). High dysphagia rates (80 with 26.7% of subjects with severe dysphagia) could also be observed in patients least affected in terms of NIHSS (NIHSS Score ≤ 8; Figure 3A and Supplementary Figure 1).

Laryngeal injury could be detected in all patients. Hereof, 5 (9.2%) presented with injury grad I, 22 (40.7%) grade II and 27 (50%) presented laryngeal injury grade III. Of 54 dysphagic patients, 46 (85.2%) showed penetration or aspiration of at least one food consistency. Aspiration of saliva could be observed in 16 (29.6%) subjects.

### Second assessment (72 h after first FEES) FEES 2

The median time between the first and second FEES examination was 73 h (IQR 70–97). Of the 49 dysphagic patients, 15 regained full swallowing function, whereas 34 still experienced some degree of dysphagia at the second FEES examination. In 5 cases, a second FEES examination could not be conducted due to decreased level of consciousness (lethargy, stuporous, and coma), or a reintubation or a determination of palliative care (Figure 1). The median FEDSS was 4 (IQR 2–6).

Nineteen patients (38.8%, median age 78 year, IQR 62–84; median NIHSS 14, IQR 8–16) showed severe dysphagia (FEDSS 4–6) with the second FEES. Among the 25% least neurologically impacted patients (NIHSS 0–4) dysphagia was observed in 7 (53.9%) of cases, with only 1 (7.7%) being severely dysphagic (Figure 3B and Supplementary Figure 1).

**Figure 4:**
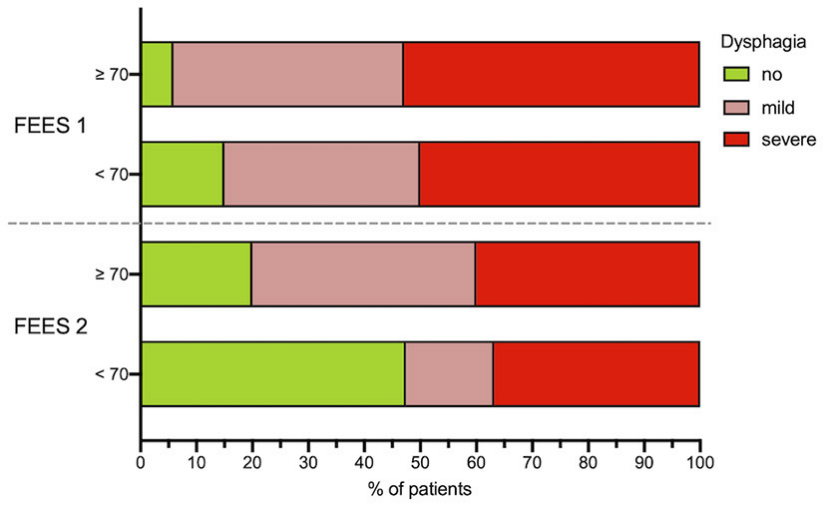
Age dependency of dysphagia. Dysphagia severity in dependence of patients age (< 70 and ≥70 years) at FEES 1 and FEES 2. Dysphagia severity is defined as no dysphagia, mild dysphagia (FEDSS 1–3) and severe dysphagia (FEDSS 4–6). *N = 5 patients did not receive second FEES due to palliative procedure. FEES, fiberoptic endoscopic evaluation of swallowing; FEDSS, fiberoptic endoscopic dysphagia severity scale, FEDSS.

Significant improvement of dysphagia frequency (χ^2^(1) = 7.49, *p* = 0.006) and dysphagia severity (*z* = −3.27, *p* = 0.001) could be detected between the first and second dysphagia assessment. In the follow-up FEES evaluation, a faster recovery from dysphagia was observed in younger patients (< 70 years) compared to patients aged more than 70 years (Figure 4). Of 34 dysphagic patients, 28 (51.9%) showed penetration or aspiration of at least one food consistency, whereas aspiration of saliva could be detected in 9 (16.7%) cases.

Twelve (22%) patients required tube feeding at discharge, as their dysphagia did not recover to a level where oral intake was possible. Total oral diet with no restrictions was present in 34 patients, while in 6 subjects total oral intake with specific texture modification and/or compensation (e.g., chin-tuck maneuver) was mandatory.

A binomial logistic regression was performed to evaluate the predictive value of following variables: age, sex, occlusion side, ventilation time, NIHSS at FEES 1, lesion location (M1, CI, I) for detection of dysphagia upon the second FEES-assessment. The binomial logistic regression model was statistically significant, χ^2^(6) = 22.28, *p* = 0.001, resulting in a large amount of explained variance, as shown by Nagelkerke's *R*^2^ = 0.536. Only, age (*p* = 0.031, OR = 1.082, CI [1.007–1.162]) and ventilation time (*p* = 0.027, OR 1.638, CI [1.058–2.535]) could be identified as significant predictors for dysphagia, while sex (*p* = 0.130, OR = 0.250, CI [0.041–1.502]), occlusion side (*p* = 0.284, OR = 2.720 CI [0.436–16.988]), NIHSS FEES 1 (*p* = 0.141, OR = 1.168, CI [0.950–1.436]) or lesion location (*p* = 0.643, OR = 0.569, CI [0.052–6.190]) were not significant (Effect of age is depicted in Figure 4).

## Discussion

This study investigated the frequency, severity, and short-term outcome of PSD in AIS patients following EVT by means of a standardized instrumental swallowing assessment. Furthermore, we tried to investigate the appropriate time point of swallowing evaluation in this specific stroke population undergoing GA.

Our data show a high frequency of PSD with 50% of patients being initially fully tube dependent (NGT).

As critical nodes of the cortical and subcortical swallowing network are located within the MCA territory, lesions in this specific brain area might have contributed substantially to the high dysphagia frequency and severity in our cohort (26, 33). For example, the insular cortex and the frontal operculum, as part of the widespread operculo-insular swallowing network, were impaired in 67 and 50% of the patients, respectively. Previous studies have demonstrated a strong association between ischemic lesions of the insular cortex and severely impaired oral intake requiring tube insertion (24, 34, 35), whereas involvement of the operculum has been linked to severe and long-lasting dysphagia (36, 37).

In binary logistic regression analysis for dysphagia, no single item was independently associated with PSD at first assessment. Interestingly, stroke severity in terms of NIHSS, which has been consistently reported as a useful predictor of PSD, showed no significant correlation in our cohort (38, 39). High dysphagia rates were also observed in patients with good neurological outcome after mechanical thrombectomy.

One possible explanation for this observation might be a remarkably high number of basal ganglia infarctions, which occur more often in patients undergoing EVT than in those treated with intravenous thrombolysis or medical treatment (18, 40). Due to the lack of collateral pathways, the basal ganglia are prone to experience infarction relatively early. Hence, patients experience less neurological sequelae, as most or all parts of the eloquent neocortex are spared. Thus, the emergence of PSD is most likely due to affection of the subcortical swallowing network, of which the basal ganglia are part (40, 41). The basal ganglia functionally connect the cerebral cortex and the thalamus, gating sensory input for motor control in deglutition. Therefore, damage to those regions can lead to disturbed oral motor control, affecting pre-dominantly the oral phase of swallowing (15, 24, 42).

Additionally, the use of general anesthesia might be considered as an additive factor contributing to the high dysphagia frequency and severity, at the first swallowing assessment as well (13). An obvious and major mechanism of dysphagia, following endotracheal intubation, is direct trauma to anatomic structures, which might more commonly arise in the acute setting of emergency diagnostic or therapeutic interventions (43). Hence, temporary laryngeal injuries like hematoma or edema, as well as hypomobility of the vocal folds, were noticed in about 90% of the respective cohort. Mechanical damage with local inflammation and edema could lead to an impairment of afferent sensory pathways, resulting in a delayed swallow response and pre-deglutitive aspiration (13, 44). Moreover, sedatives and/or various neurotropic medications are known to an impact on swallowing, either centrally or peripherally (11, 13).

At the second FEES assessment, improvement of swallowing could be observed in 30% of the patients recovering completely from dysphagia, whereas the remaining patients were still dysphagic In light of these findings, it can be assumed that dysphagia following EVT is not a short-term symptom which rapidly recovers (e.g., drug effects have vanished). In this context, insult to the brain before EVT might bear potential for lingering dysphagic symptoms. Infarction in the basal ganglia or internal capsule is more commonly observed in the setting of highly effective reperfusion therapy (18, 41). As those brain regions play a vital role in the subcortical swallowing network recovery, they might have an impact on dysphagia prevalence and recovery. However, future studies are warranted to investigate the impact of lesion location on dysphagia in patients undergoing EVT. Regarding the recovery of dysphagia, older patients (>70 years) were particularly affected by persisent swallowing dysfunction, in contrast to the younger subgroup. At this time point, age and duration of intubation were significantly associated with swallowing disturbance. Age-related changes of swallowing—termed as presbyphagia—are usually compensated for and clinically in-apparent. However, they can impair the ability to compensate for disease-related swallowing dysfunction—in this case stroke and ICU treatment—impacting dysphagia outcome and recovery (45, 46). Likewise, robust data exists on intubation duration, as a significant risk factor for dysphagia (16, 47) due to pharynolaryngeal lesions caused by the endotracheal tube (13). Consequently, younger patients and those with shorter intubation time recovered faster from dysphagia.

Recommendations to assess dysphagia in acute stroke patients are widely implemented in clinical guidelines (2, 5). However, they are not narrowly tailored to this specific stroke treatment (EVT) setting including post-extubation management (20). Hence, it remains unclear when and how to evaluate swallowing post-extubation in patients undergoing EVT.

Our observation does not support the current general practice of delaying swallowing evaluation by at least 24 h post-extubation, assuming spontaneous improvement over time (47). Rather, we recommend a timely assessment of swallowing function after extubation, regardless of possible consequences of ICU procedures (e.g., endotracheal intubation, prolonged ventilation, and sedation) on swallowing function, as in 50% of cases patients were safe to begin oral intake under dietary restrictions. Previous studies have shown that delays in PSD evaluation were associated with stroke-associated-pneumonia, with an absolute risk of pneumonia incidence of 1% per day of delay (48).

Based on our preliminary findings, dysphagia assessment could be conducted as soon as 2 h following extubation. Early dysphagia assessment including FEES evaluation, which can be performed at the bedside on the ICU, where EVT patients are mostly treated initially, allowed for early initiation of an oral diet. Interestingly, patients starting an early oral diet showed significantly lower pneumonia rate than those with NGT feeding. This could be due to the stringent use of FEES to define the safest feeding route, which in contrast to clinical swallowing examination, is known to offer higher specificity in diet prediction, as well as higher sensitivity in identifying aspiration events (1, 49, 50). As a result, fewer patients were tube (NGT) dependent, which in turn has been identified as a potential risk factor for pneumonia, due to its potential to contribute to infection by promoting oral-pharyngeal colonization or aspiration (22, 48).

Additionally, patients >70 years should be carefully monitored by the multidisciplinary stroke team, and particularly an SLT, as recovery of dysphagia is prolonged in this cohort. Even those patients with mild neurological deficits can suffer from long-lasting dysphagia, as age-related changes can delay the recovery of swallowing function. Nevertheless, about 80% of dysphagic patients regained full oral diet by the time of discharge.

## Limitations

This exploratively preliminary single-center observational study has several limitations, including its small sample size and the lack of a control group undergoing EVT in conscious sedation. Additionally, due to the low number of basilary artery occlusion we could not provide specific data on the nature of dysphagia in this specific subgroup. Therefore, our observations should be validated in a larger multicenter study.

## Conclusion

Poststroke dysphagia is a frequent finding, both immediately within 24 h after extubation, as well as in the short-term course. Our observations indicate that swallowing assessment can be conducted timely after extubation, as it provides valid information regarding the presence of post-stroke dysphagia and its severity. According to our findings, there is no need for delaying evaluation of swallowing function, as more than 50% of dysphagic patients were able to start with an oral diet, under certain food restrictions.

In conclusion, we strongly recommend the use of instrumental swallowing diagnostics to determine the safest feeding route, due to its higher accuracy with respect to diet recommendation and the detection of aspiration. As patients with advanced age and prolonged intubation showed delayed recovery of swallowing function, we also strongly recommend that these patients be closely monitored by an interdisciplinary team.

## Data availability statement

The raw data supporting the conclusions of this article will be made available by the authors, without undue reservation.

## Ethics statement

The studies involving human participants were reviewed and approved by Goethe University Hospital Frankfurt (approval no. 19-262). The patients/participants provided their written informed consent to participate in this study.

## Author contributions

SL, CF, and SR involved in drafting the article or revising it critically for intellectual content. All authors involved in conception and design of the study, or acquisition of data, or analysis and interpretation of data and approved the final submitted version.

## Conflict of interest

The authors declare that the research was conducted in the absence of any commercial or financial relationships that could be construed as a potential conflict of interest.

## Publisher's note

All claims expressed in this article are solely those of the authors and do not necessarily represent those of their affiliated organizations, or those of the publisher, the editors and the reviewers. Any product that may be evaluated in this article, or claim that may be made by its manufacturer, is not guaranteed or endorsed by the publisher.

## Supplementary material

Supplementary Figure 1 Distribution of the NIHSS and FEDSS at FEES 1 and FEES 2. “0” represents patients without dysphagia.

## Abbreviations

AIS: acute ischemic stroke
EVT: endovascular treatment
FEES: fiberoptic endoscopic evaluation of swallowing
FEDSS: fiberoptic endoscopic dysphagia severity scale
GA: general anesthesia
IQR: interquartile ranges
IVT: intravenous thrombolysis
LVO: large vessel occlusion
NIHSS: national institutes of health stroke scale
MT: mechanical thrombectomy
mRS: modified Rankin scale
PSD: post stroke dysphagia
SLT: speech and language therapist.
