# Supplementary figures and images for: Dysphagia assessment in ischemic stroke after mechanical thrombectomy: When and how?
Source: Front Neurol. 2022 Nov 23;13:1024531. doi: 10.3389/fneur.2022.1024531 (PMC9726734; doi:10.3389/fneur.2022.1024531)

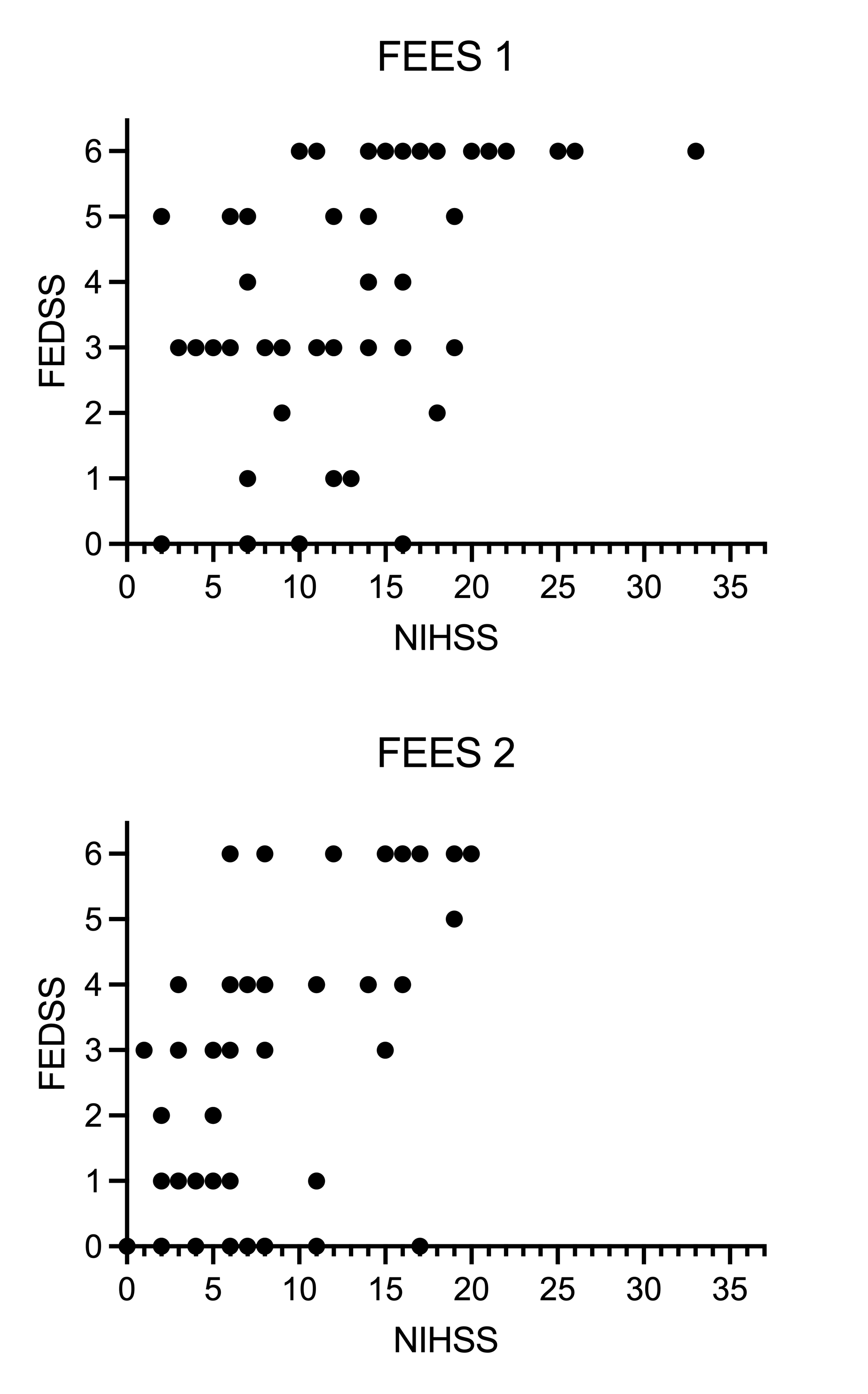

Supplement: Supplementary Figure 1 — Distribution of the NIHSS and FEDSS at FEES 1 and FEES 2. “0” represents patients without dysphagia. [file Image_1.TIFF]
